# Flower Development as an Interplay between Dynamical Physical Fields and Genetic Networks

**DOI:** 10.1371/journal.pone.0013523

**Published:** 2010-10-27

**Authors:** Rafael Ángel Barrio, Aurora Hernández-Machado, C. Varea, José Roberto Romero-Arias, Elena Álvarez-Buylla

**Affiliations:** 1 Departamento de Física Qumica, Instituto de Física, Universidad Nacional Autónoma de México, México D.F., Mexico; 2 Department of Structure and Constituents of Matter, Facultat de Física, Universitat de Barcelona, Barcelona, Spain; 3 Instituto de Ecología, Universidad Nacional Autónoma de México, México D.F., Mexico; University of Milano-Bicocca, Italy

## Abstract

In this paper we propose a model to describe the mechanisms by which undifferentiated cells attain gene configurations underlying cell fate determination during morphogenesis. Despite the complicated mechanisms that surely intervene in this process, it is clear that the fundamental fact is that cells obtain spatial and temporal information that bias their destiny. Our main hypothesis assumes that there is at least one macroscopic field that breaks the symmetry of space at a given time. This field provides the information required for the process of cell differentiation to occur by being dynamically coupled to a signal transduction mechanism that, in turn, acts directly upon the gene regulatory network (GRN) underlying cell-fate decisions within cells. We illustrate and test our proposal with a GRN model grounded on experimental data for cell fate specification during organ formation in early *Arabidopsis thaliana* flower development. We show that our model is able to recover the multigene configurations characteristic of sepal, petal, stamen and carpel primordial cells arranged in concentric rings, in a similar pattern to that observed during actual floral organ determination. Such pattern is robust to alterations of the model parameters and simulated failures predict altered spatio-temporal patterns that mimic those described for several mutants. Furthermore, simulated alterations in the physical fields predict a pattern equivalent to that found in *Lacandonia schismatica*, the only flowering species with central stamens surrounded by carpels.

## Introduction

Undifferentiated cells are identical at many different scales, they share not only the same DNA, but also the same genes and the same overall gene regulatory networks (GRN), that in turn underlie heterogeneous expression patterns for each gene in space and time during development. Hence, multi-gene configurations are established during development, and cells attain specific fates at particular sites and times, in response to signals that are dependent on their position, and/or their cell lineage. Experimental results suggest that in plants cell differentiation strongly depends upon positional information [1], but it is likely that cell fate is the result of a dynamic interplay between positional information and intracellular GRN dynamics [2]. Nonetheless, understanding how positional information is generated and maintained, as well as how such information is coupled to intracellular GRN dynamics, are key to understanding pattern formation during development.

Cell fate can be determined by a single GRN if it presents multiple attractors, each determining the expression profile (expression state of all the genes within the GRN), that is characteristic of each cell type. Recently, it has become possible to postulate GRN models grounded on experimental data. Such models have been successful at discovering developmental modules or sub-networks able to recover and predict multi-gene expression profiles observed in cell types, thus providing a dynamical mechanism to understand how different cell types are attained, given a fixed GRN topology that should be present within all the cells [3]–[5]. However, multicellular models that explicitly incorporate cell-cell coupling mechanisms to generate a meta-GRN model, in which the spatial and temporal dynamics of cell-fate attainment can be followed are only starting to appear. For example, Benítez and collaborators [2] were able to recover spatial cell patterns observed in *Arabidopsis thaliana* epidermis by coupling intracellular GRN via diffusion of some of the network components according to available experimental data.

Undoubtedly, cell differentiation is a complicated choreography that should involve intricate interactions between intercellular communication mechanisms and intracellular processes that regulate gene expression, without a central controller or principal choreographer. Instead, cell differentiation and morphogenesis take place in structures with specific physical characteristics that establish fields that, at least, should reinforce positional information also emerging from molecular mechanisms that couple and feedback from the dynamics of intracellular GRNs during cell differentiation. The importance of such physical fields and mechanisms has recently received special attention providing a new approach to developmental biology. For example, a recent study [6] has demonstrated that alterations of the stress distribution, that determines the patterns of microtubule orientations in *Arabidopsis thaliana* shoot apical growing cells, modifies morphogenesis in a predictable way. However, this and similar papers have not explicitly coupled such physical fields to the dynamics of the GRN underlying cell-fate determination.

In this paper, we focus on a previously characterised GRN that underlies floral organ primordial cell specification during early stages of flower development [4]. We use this example in order to illustrate a more general approach to couple GRN dynamics across tissues with a single physical field that provides positional information. The studied GRN has been shown to recover the multigenic expression profiles observed in the four main types of primordial cells established during early stages of flower development (see for instance, [7]–[10]).

In contrast to animals, plant morphogenesis takes place during the entire life cycle from groups of undifferentiated cells called meristems. Two main meristems remain active during the whole life-cycle of plants: the shoot apical meristem (SAM) and the root apical meristem (RAM). From the former, the inflorescence meristem arises upon the transition to flowering and in its flanks flower primordia are formed.

During early floral development, the floral meristem is subdivided into four concentric regions of primordial cells that will eventually form the floral organs that from the outside to the centre are: sepals, petals, stamens and carpels [10]. The spatial pattern of flower morphogenesis is widely conserved among the 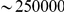 flowering plant species and thus a robust and generic underlying mechanism is expected, but not well understood up to now. The only exception is *Lacandonia schismatica*, whose flower presents a homeotic inversion with central carpels surrounded by stamens.

The now classical ABC model of flower development [11], [12] establishes the necessary combinatorial gene functions for sepal, petal, stamen and carpel specification [8]. The ABC model proposes that class A genes alone are responsible for the development of sepals, but act together with class B genes to specify petal development. Class C genes alone are responsible for specifying carpel development, but act together with class B genes to determine stamen development. However, this model does not explain how such combinatorial gene functions are spatio-temporally established during flower development.

Cell differentiation thus involves at least two aspects. First, a physical field is required to break the symmetry of the spatial domain into different regions in which distinct sets of transcription factors are expressed and exert their function. Therefore, a phase-field model of the kind used in the physics of free boundary problems [13] could be used to model physical fields in any developmental system. Second, a GRN responding to the physical field, and consequently able to reach different attractors (fixed gene configurations) depending on the cell position in space. In this paper we aim to showing that such interplay between a physical field and the dynamics of the GRN is sufficient to recover a morphogenetic pattern that resembles that observed during early flower development. The first component involves a macroscopic field, while the second aspect implies modelling the GRN dynamics that occurs at a microscopic scale. Physical fields may be of various types and they could be sensed by morphogens, such as auxins in plants. In fact meristems and primordia of lateral organs are formed in places where there is a peak of auxin concentration [14], which seems to trigger the production of undifferentiated cells. Other chemicals, as cytokinins, have been proposed to start the formation of the meristem, which paradoxically are substances that inhibit cell proliferation.

This paper is organised as follows: In the next section we describe in detail the physical field model that is used to generate the spatio-temporal information. Then, we postulate a simplified version of the flower organ identity GRN and the mechanism by which the GRN is coupled to the macroscopic physical field. In the third section we present results from numerical calculations from the model that couples the GRN dynamics and the physical field. Our results suggest that such coupled dynamics is sufficient to recover a geometrical distribution of the flower organ primordia that resembles that observed during early flower development. In order to validate the model we analyse all possible mutations predicted by this model and compare results with patterns of previously studied mutants, or provide predictions for those which have not been studied and for the effects of altering the physical field or the shape of the meristem.

## Methods

### Spatio-temporal Model

Experimental evidence suggests that the flower meristem arises at a position where a peak of auxin concentration is established. Around such maximum a Gaussian auxin distribution is generated. Such morphogen distribution underlies the geometry of the early forming flower meristem, which grows isotropically around the auxin peak at early stages of flower development [15], [16]. Therefore, it is sensible to assume a spherical meristem to specify the spatio-temporal domain in order to model cell differentiation dynamics at that stage of development. In the spirit of the ABC model we need to obtain a region in space where A dominates and another region where C dominates. This can be accomplished by defining a parameter 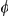 that separates the inner region, where 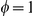, from the outer region, where 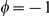. These regions will eventually be separated by a sharp interface because A and C genes repress each other [11]. There are similar systems in which it is assumed that minimising the bending energy of the interface is the main driving force of the dynamics of this parameter [17]–[19], as when studying vesicle formation, or the shape of red blood cells and other lipid bilayers. This bending energy contains contributions of both, the mean curvature and a spontaneous curvature that could depend on the spatial position of each point or cell within the modelled domain. Also in the spirit of the ABC model, we have to consider another parameter 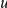 that represents the B component, and that should be coupled to 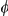 throughout the space. The values of these parameters in all the space define two fields. The “phase-field” 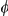 is then coupled to field 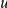 through a “spontaneous curvature-like” term. Since we are restricting ourselves to the first stages of cell differentiation, we may assume that in the time scale when the fields evolve, the size of the domain (the meristem) remains constant, and that both fields are conserved, that is, the total “mass” and the area surrounding the volume of the system are constant. Therefore, we propose a physical system whose free energy density is
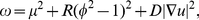
(1)where 
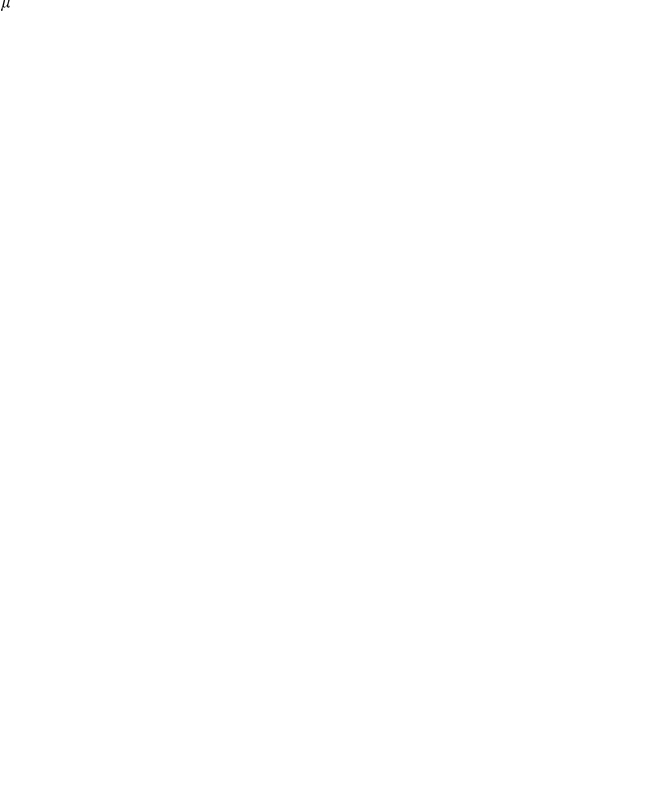
 is [19]

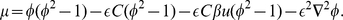
Here 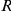 is the potential for field 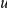, and should depend on the expression of the GRN locally, 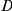 is related to the energy cost of creating a 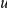 profile, and 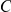 is the local spontaneous curvature, 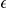 is a parameter that measures the interface sharpness, and 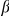 is a parameter that is proportional to the strength of the interaction between the fields.

Integrating over the volume ones defines
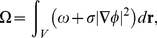
(2)whose variations 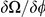 and 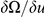 give the dynamic equations for the evolution of the fields 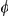 and 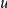, respectively
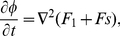
(3)

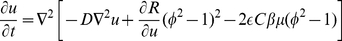
where
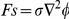


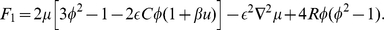



Notice that the Laplacian assures the conservation of both fields. The parameter 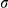 is a Lagrange multiplier that assures the conservation of the area [19], and it is determined by calculating the area 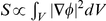 and demanding that 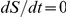. Using Eqs. 3 we obtain
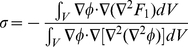
(4)where the integrals are over all the volume. One could eventually relax these conservation laws if one is interested in including cell proliferation with the subsequent growth of the domain in which the fields act.

### Coupled Genetic Network

**Figure 1:**
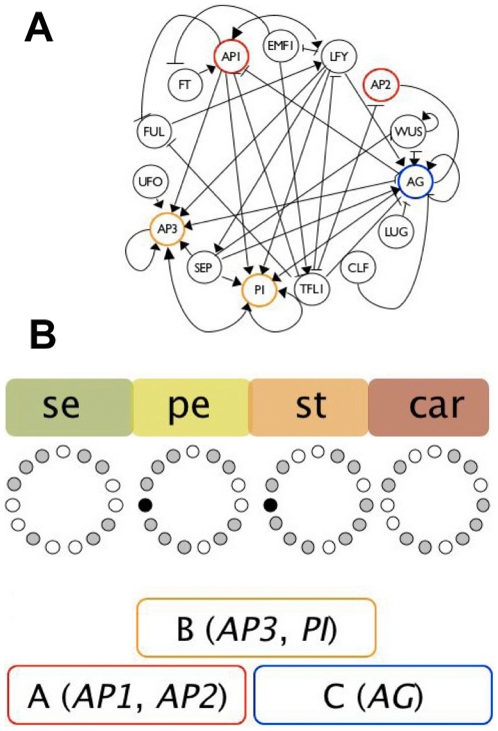
Gene regulatory network (GRN) model underlying cell fate determination during Arabidopsis thaliana floral organ determination during early flower development. (A) GRN with interactions inferred from experimental data (according to [4], [8]). The genes from the ABC model are highlighted in the GRN: A-genes (red), B-genes (yellow) and C-genes (blue). (B) Attractors or steady state gene configurations of the GRN model that match gene expression profiles expected by the ABC model are provided. Expressed genes for each attractor are represented as grey circles, while non-expressed ones correspond to white circles, the black circle corresponds to a gene (UFO) that can be either expressed or not expressed in the petal and stamen attractors, thus yielding two attractors for each petal and stamen primordia cell-type (taken from Ref. [9]).

In Fig. 1(a) we show a GRN network, grounded on experimental data, proposed in Ref. [9]. It contains 15 genes, wherein their interactions were formalised as logical functions. Five of these are grouped in the so-called A, B, and C genes, whose combinations are necessary for floral organ cell specification [11]. The ABC model establishes the combinatorial gene functions necessary for cell specification during early flower development. A-type genes (AP1 and AP2) are necessary for sepal identity, A-type together with B-type (AP3 and PI) for petal specification, B-type and C-type (AGAMOUS) for stamen, and the C-type gene (AG) alone for carpel primordia cell specification. In Fig. 1(b) we show these genes grouped in coloured boxes and also some of the attractors to which the 15-gene GRN model converges [4].

A recent publication showed that a stochastic version of the floral GRN model recovers a temporal sequence of cell-fate attainment that mimics that observed in most flowers: once sepal primordia are determined, then petal are differentiated next, and then stamen and carpel primordia are determined [10]. However, the mechanisms that underlie both the temporal and the spatial robust patterns observed in *Arabidopsis thaliana*, which are shared by the great majority of flowering plants, remain largely unexplored. This is true for practically any biological system, and models of coupled cellular GRN that consider the role of physical fields are only starting to be developed [20], [21].

**Figure 2:**
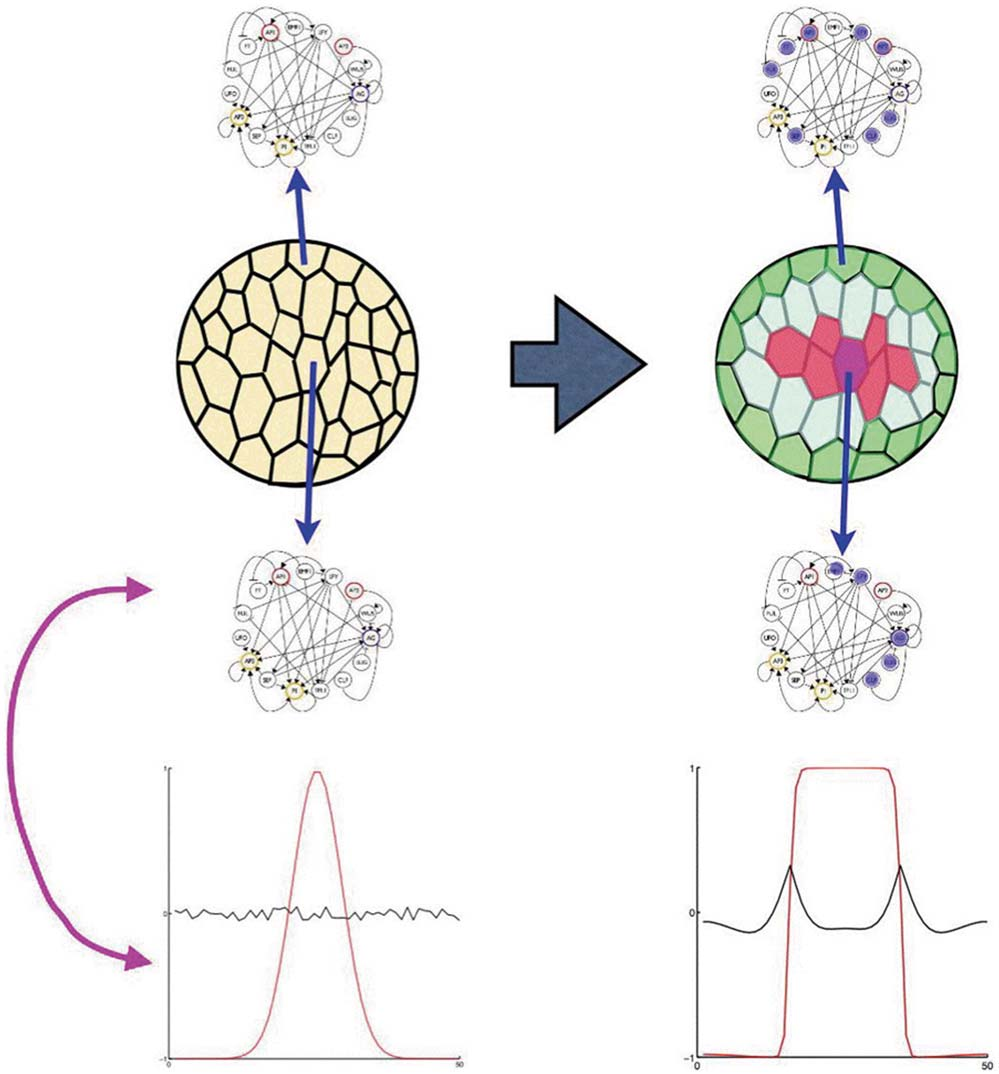
Schematic representation of the proposed interplay between the intracellular gene regulatory network (GRN) dynamics and physical fields that lead to the spatio-temporal patterns of cell types. On the left hand side we show the undifferentiated cells in yellow, each having identical GRNs and gene configurations. Such configurations correspond to the state of activations of each of the genes within the GRN. The profile of the initial field 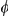 is shown in red and the field 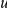 is shown in black. As time runs, there is a reciprocal interaction between the fields and the GRN, represented by a purple line. On the right hand side we show the state of the fields and the GRNs within cells after some time. The GRNs respond to the external fields, which provide positional information, and thus attain different attractors depending on the value of the fields at each cell position. These attractors or gene configurations are represented by showing activated genes in blue, while the colours of the cells indicate different lineages and fates.

The mechanism we propose to produce cell differentiation is a dynamic coevolution between the macroscopic fields 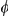 and 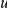 and the genetic networks in each cell. The idea is depicted schematically in Fig. 2. Initially there is a chemical signal, represented by a gaussian distribution of some substance that regulates the initial state of all GRN within the floral meristem cells. This triggers the chemical reactions described by our phase field model and also induce each GRN to attain a different attractor in a coevolving way, that is, depending on the values of the external fields in space and time, different attractors are chosen, and this in turn reinforces the changes of the external fields through a reciprocal interaction. This mechanism will lead to produce cells of different types disposed in a geometrical arrangement of concentric rings in the early stages of the flower development. These eventually produce the four mature organs of the flower differentiated in concentric rings

For that we need to describe how the GRN of each cell responds to the external physical fields proposed in the last section. We examine the network of Fig. 1(a) and simplify it as much as possible, preserving the functional loops (see Ref. [22]) that are sufficient to recover the ABC patterns. We observe that the difference of the attractors that correspond to petal and sepal primordial cells only differ on the state of the gene 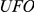, and the same is true for the floral organs that produce the gametes (stamens and carpels). This gene is interacting with the network only as an activator of 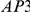, and therefore its function could be regulated directly by the field 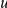. A similar situation applies to the gene 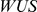 (a key gene during the early stages of flower development), which regulates only the actions of 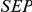 and 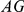, the former being an activator of 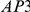 and 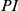. The presence of a feedback loop makes us expect that the actions of this gene could be controlled externally by the field 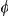 just as well.

**Figure 3:**
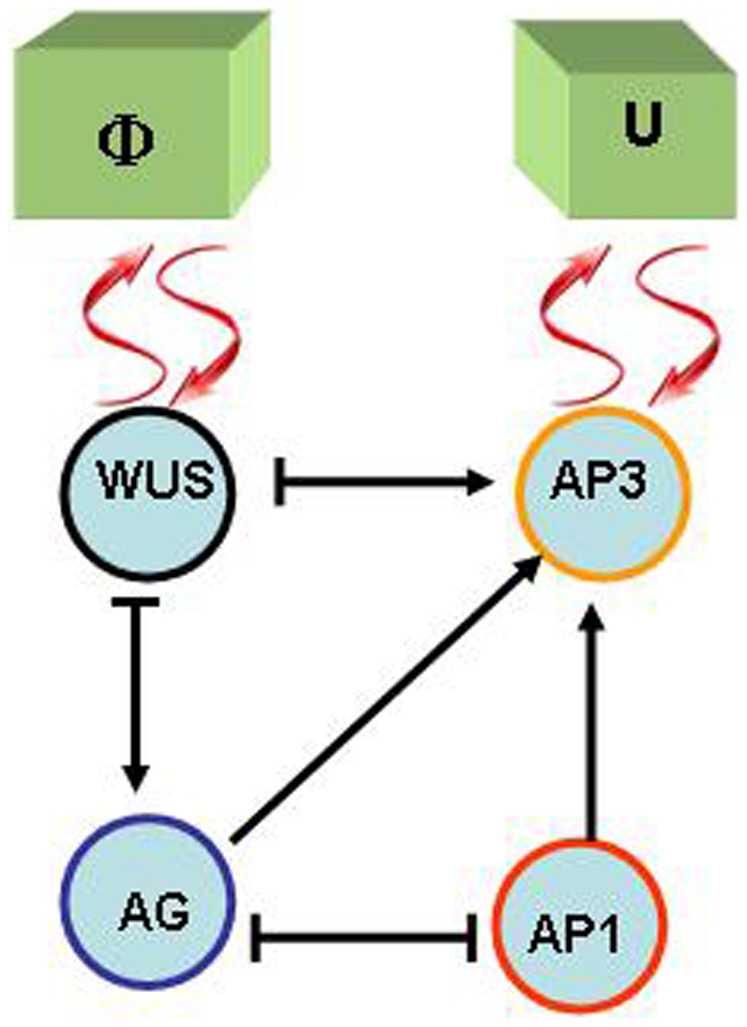
Simplified gene regulatory network. An arrow indicates an activation, a bar at the end of an edge indicates an inhibitory interaction. Wavy arrows indicate two-way interactions between the GRN and the macroscopic physical fields 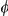 and 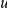. This model is also in agreement with recent accounts [24] of the molecular genetics of flower development and is congruent with the original GRN shown in Fig. 1.

The resulting GRN is shown in Fig. 3. Comparisons of this simple network with the original one shows that we have conserved all the interactions among genes associated with the ABC functions. The interactions shown in Fig. 3, either activators or inhibitors, allow us to determine the resulting binary state of all genes, given the state configuration at a certain time. If 1 corresponds to a gene that is expressed, and 0 stands for a non-expressed gene, the network of Fig. 3 follows the logical rules:
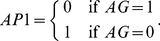
(5)

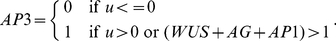
(6)

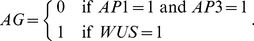
(7)

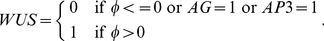
(8)


Observe that the fields 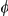 and 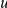 affect the expression of 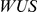 and 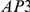 respectively, as indicated in the figure. From these logical rules we have built the truth tables for this network and found that for the 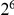 possible initial conditions the GRN has only four attractors, and each one of them can be assigned to the gene expression profiles of one of the four types of floral organ primordial cells, namely
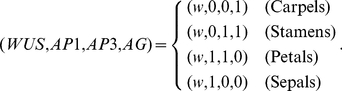
(9)This assignment was made in the spirit of the ABC model, associating the activation of 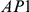 to A, of 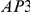 to B and the activation of 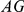 to C.

If we consider a three dimensional space filled of cells, each with the same GRN, then the local values of the fields 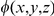 and 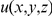 will dictate the state of the GRN in each cell. Additionally, the dynamics of the fields (Eqs. 3) could be modified by the GRN through the potential 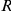 in Eq. 1. If one uses a potential for the 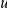 with two wells, for instance 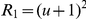 and 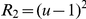, and defines
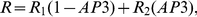
(10)then the attractor for 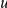 is determined by the state of 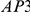.

We finally achieve the coupling between the macroscopic fields that provide the spatial information, and the GRN that incorporates key regulators of cell differentiation, assuming that the chain of chemical reactions that lead to the final specification of different organ primordia follows from this interaction directly.

## Results

### Numerical Solution

Normally, the shoot apical meristem has cylindrical shape. This allows us to assume that there are no variations of the fields around the axis of symmetry. Therefore, the domain is totally defined by its two dimensional shape along any plane and we can integrate the dynamical equations of the model in a two-dimensional domain by defining the shape of the initial conditions for 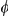 according to its orientation with respect to the symmetry axis in three dimensions. In all the numerical calculations the domain was a square grid of 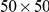, in which each pixel represents a cell. Zero-flux boundary conditions on the border of the square domain are the appropriate ones to avoid spurious results from periodic boundaries or finite size effects. We used a simple Euler method, which proved extremely stable for a time step 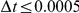. Initially 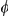 was taken as a Gaussian centred in the domain and with a reasonable width of 8 to 30 cells. The formation of a phase boundary is a condition *sine qua non* the whole process of differentiation succeeds. The initial 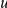 was taken as a small random noise around a constant value near zero. This is represented schematically on the bottom left hand side of Fig. 2.

One has to recognise that the time scale in which the macroscopic fields change is much smaller than the time scale in which the genes respond to external signals, since the latter process involves the production of proteins and other complicated biochemical processes. In order to take this into account in our numerical calculations, we followed the dynamics of the macroscopic fields with a time step 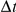, and every lapse 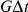 we called for the action of the GRN, where 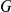 was estimated to be of the order of 100. These values were estimated by taking into account the temporal studies of flower development of Ref. [12] and the results barely change within a reasonable range of values.

**Figure 4:**
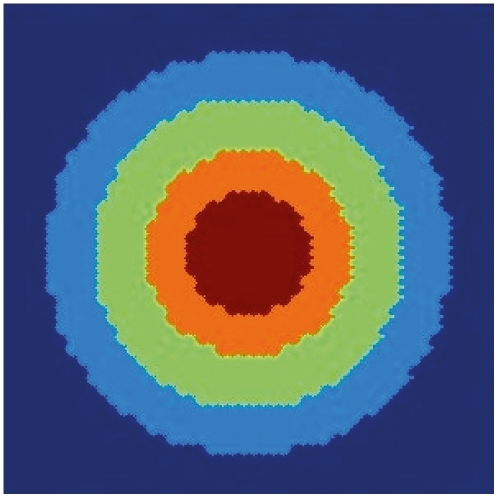
Simulated spatial arrangements of primordial floral organ for wild type plants. Numerical results for the spatial arrangement of gene configurations in a two dimensional domain that corresponds to a plane perpendicular to the axis of symmetry of the meristem. Carpels in red, stamens in orange, petals in light green and sepals in light blue. The undifferentiated cells are in dark blue.

If we assume that the two-dimensional spatial domain is a plane perpendicular to the axis of symmetry of the meristem, then we expect the appearance of concentric rings of cells each ring with contrasting gene expression stable profiles that correspond to those observed in real floral primordia. In Fig. 4 we show the results of a calculation with 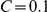, 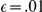, 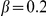, 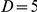 and 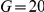. Observe that the model yields a spatial disposition of concentric rings that mimic those observed in real floral meristems. Notice that the plot is not smooth, since each pixel has a specific gene configuration.

**Figure 5:**
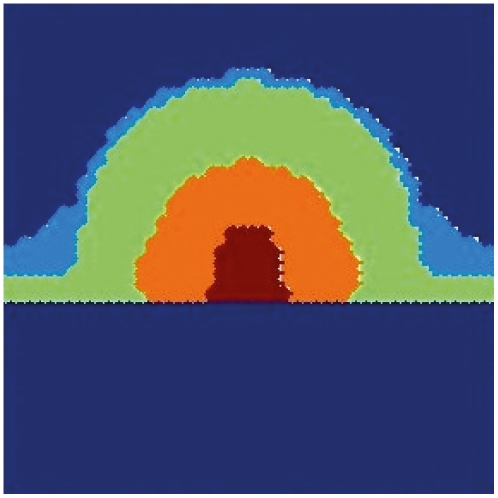
Simulated spatial arrangements of primordial floral organ for wild type plants. Numerical results on a plane containing the axis of cylindrical symmetry of the meristem in an early stage of development. Carpels in red, stamens in orange, petals in light green and sepals in light blue. The undifferentiated cells are in dark blue.

We have also investigated the spatial arrangement achieved in a plane that contains the axis of symmetry, that is, a cross section of the meristem. The results are shown in Fig. 5. Observe that the position of the cells with the gene profiles observed in each one of the primordial floral organs corresponds to those observed experimentally. Furthermore, it is remarkable that the section corresponding to sepals (in light blue in the figure) emerges in locations where the curvature has experienced a major change. This type of bulging during early stages of sepal development is observed in real floral meristems (see for instance Ref. [16]).

### Mutants

In order to validate our modelling approach, we have simulated mutants. With the complete GRN model we can investigate the various mutants that the model can predict. These variations are found when some of the GRN components are turned “off” or are ectopically turned “on”. In this paper we considered only the gene mutations for the components in Fig. 3.

**Figure 6:**
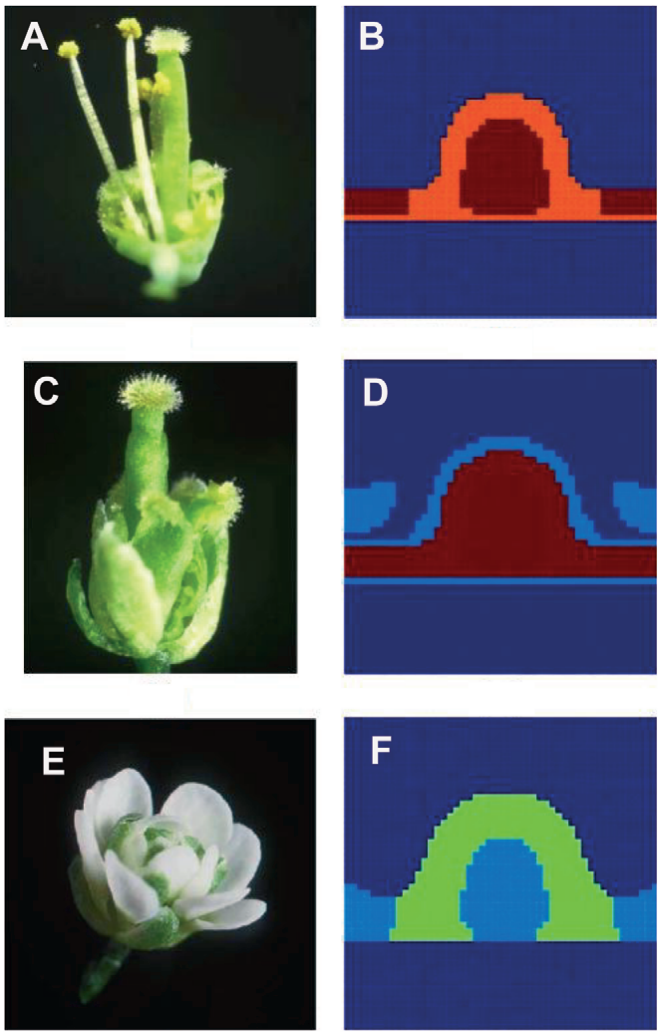
The model recovers spatial configurations that resemble those of ABC mutants when these are simulated. (A) A-mutant flower showing a homeotic conversion of sepals and petals into carpels and stamens, respectively. (B) Simulated A-mutant with 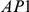 set to 0, showing a spatial arrangement of configurations corresponding to stamens and carpels similar to that shown in (a). (C) B-mutant flower with a homeotic conversion of petals and stamens into sepals and carpels, respectively. (D) Simulated B-mutant obtained by setting 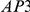 to 0, showing a spatial arrangement of configurations corresponding to sepals and carpels similar to that shown in (b). (E) C-mutant flower with a homeotic conversion of stamens and carpels into sepals and petals, respectively, and a loss of flower meristem determinacy. (F) Simulated C-mutant obtained by setting 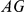 to 0, showing a spatial arrangement of configurations corresponding to sepals and petals similar to that shown in (E). Carpels in red, stamens in orange, petals in light green and sepals in light blue. The undifferentiated cells are in dark blue.

For instance, if 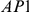 is set to zero, one gets the A-mutant type. Likewise, if 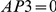 one gets the pattern observed in B-mutant type, and if 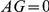 the pattern observed in the C-mutant type is recovered. The results of the calculations for these three mutants are shown in Fig. 6, together with their experimental realisations. These mutations are the main ones predicted by the classical ABC model. Our model recovers the observed spatial distributions of the gene configurations when these mutants are simulated. But our model is also able to make novel predictions.

**Figure 7:**
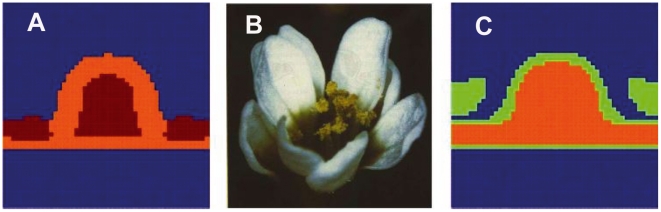
The model recovers spatial configurations similar to those observed in over-expression lines of 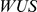 and 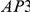. (A) Spatial configuration obtained by setting 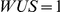. (B) Flower of an over-expression line for AP3 in which petals, stamens and petals are observed (taken from Ref. [23]). (C) Simulated B function over expression obtained setting 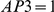. Carpels in red, stamens in orange, petals in light green and sepals in light blue. The undifferentiated cells are in dark blue.

For instance, if 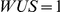 one gets a flower very similar to the A-mutant type, as shown in Fig. 7(a), that is, a flower with carpels and stamens only. Notice that the space distribution of the organs is slightly different from the true A-mutant. Also, if one sets 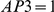 [23], a similar flower structure to that observed when this B gene is over-expressed is recovered: a structure that only has configurations corresponding to stamens and petals (Fig. 7(b) and (c)).

**Figure 8:**
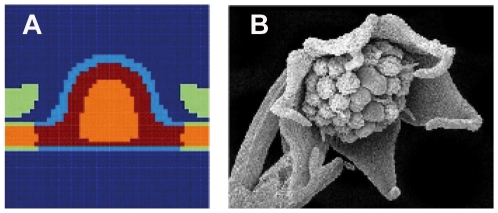
Lacandonia-like mutant predicted by the model. (A) Inverted pairs mutant type (Pe,Se, Car,St), obtained reversing the potential 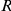. Carpels in red, stamens in orange, petals in light green and sepals in light blue. The undifferentiated cells are in dark blue. (B) Photograph of the flower of *Lacandonia schismatica*. Note that in the perianth of this flowering species sepals and petals are not distinguished, and it has six so-called tepals, yet its reproductive organs are inverted, as those of the simulated mutant.

As our model couples the intracellular GRN dynamics with the physical fields, another way to cause alterations in the spatial arrangement of genetic configurations characteristic of different cell types, is by altering the potential functions. For example, an interesting altered pattern is recovered by reversing the sign of the potential 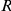 of 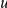 (interchanging 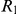 and 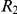 in Eq. 10). Such reversion produces an inversion in the location of organs within two pairs: sepals and petals and stamens and carpels (see Fig. 8(a)). It is interesting that in natural and experimentally created mutants it is very rare to observe inversions between individual adjacent organs. Our model suggests that a possible explanation to this observation is that the physical fields, that are important for breaking the symmetry of floral meristems, are strongly constrained. This would in turn explain the fact that the simulated alteration has not been observed in most previous experimental and natural homeotic mutations. In other words, for some yet not understood reason such constraints have not been broken in real systems, as we have done so in our simulations with altered fields. In the present model it is not straightforward how one could justify such a failure of 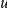, since it would imply several alternative components of the signal transduction pathways that link the physical fields to the GRNs and these are not explicitly considered in the present version of the model. Such failures could also imply alterations on the physical fields themselves.

It is also interesting that in *Lacandonia schismatica*, (see Fig. 8(b)) the only flowering plant with inverted carpels and stamens, the underlying molecular mechanism of such reversal seems to imply the shift of the domain of expression of one of the B genes to the flower centre. Such a shift could, in fact, be due to an alteration of the physical field itself, or the way in which this field is perceived by the cells at different moments during flower development. More detailed studies would be needed to specifically model such alterations as a result of modified fields and positional information perceived by particular genes.

### Effects of the Geometry of the Apical Meristem

**Figure 9:**
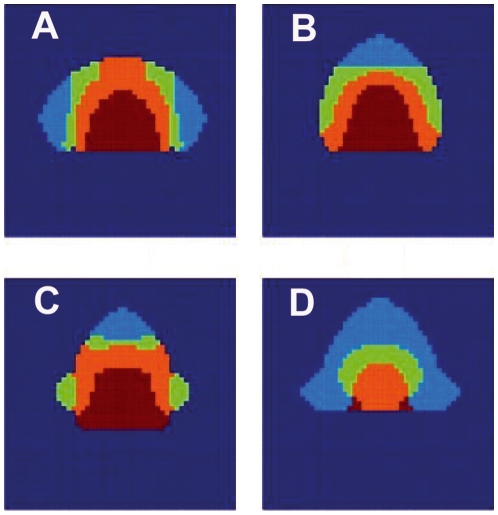
Varying the geometry of the meristem. The genetic expression on a circular domain is shown in Fig. 4, the same type of calculation gives different accommodation. The colour code is: Carpels in red, stamens in orange, petals in light green and sepals in light blue. The undifferentiated cells are in dark blue. (A) domain with two sharp lateral edges. (B) Domain with an acute apical end. (C) Domain with acute apical end and two lateral round wings. (D) Domain with three acute edges.

Given that we have a model of GRNs coupled in an explicit spatial domain which dynamics feedback to physical fields, we may investigate the influence of the geometry of the spatial domain in flower morphogenesis. We have addressed how the spatial disposition of floral organs is altered when the geometry of the apical meristem is not spherical. In order to do this we have designed a program in which we define a domain of certain shape and impose zero-flux boundary conditions at the border. Then we made a series of calculations starting with identical initial conditions and changing the shape of the domain. In Fig. 9 we show some examples of these calculations. Notice that if the domain is not approximately spherical, serious deviations of the normal disposition of the organs can occur. This can be considered as a further prediction of the model, that could in principle be tested experimentally if such alterations in flower meristem geometry could be achieved. In any case, these simulations suggest that alterations in floral organ disposition may be obtained without invoking genetic mutations in the floral organ GRN.

Finally, we have performed numerous calculations exploring the effect of varying the various parameters of the model within reasonable ranges, and have verified that the main results of the model presented in this section are robust and trustful.

## Discussion

We have presented a spatio-temporal model in which intracellular GRNs are coupled by physical fields, which provide spatial information to cells and thus trigger cell differentiation. This model includes a specific coupling between physical fields with the cellular GRNs. This coupling is dynamical, and can be regarded as a description of the dynamical coevolution between chemical changes in the intercellular environment and gene expression within the cell.

We applied our model to study floral organ specification during early *Arabidopsis thaliana* flower development. In this case, an experimentally grounded GRN is available [4]. In this paper we have been able to recover a spatial distribution of the four floral organ primordia that mimics that observed in real flowers. Furthermore, our model is able to predict the various flower arrangements found in wild type flowers and those observed in mutants, as well as changes caused by modifications of the shape of the meristem, or alterations in the physical fields. These predictions suggest that one can design experiments to modify the shape of the meristem during the early stages of flower development, in the same spirit of the ones performed in Ref. [6], or alter the physical fields and expect alterations in flower organ disposition.

The recovery of patterns similar to those observed in actual wild type flowers, including the appearance of sepal primordia as bulges in the outer part of the floral meristems, as well as the spatial patterns of genetic configurations observed in mutants, serve as a validation of the overall assumptions of the model and provide some new predictions. Nonetheless, it is likely that more detailed GRN and physical field models will be required to provide more specific predictions. The fact that our results are fairly robust to alterations in the parameters, suggest that the overall coupling of GRN and physical dynamics, proposed here, very likely incorporates key aspects of flower morphogenesis and provides a plausible hypothesis for the emergence of positional information during cell patterning.

In this paper we have presented results keeping the size of the meristem constant. In actual flower development it is undeniable that this is not the case and our calculations should be regarded as a dynamical process in which cell differentiation and proliferation occur, and at early stages yield domain growth and later on balance each other when a final domain size is attained. It is known that the different whorls of organs appear in the meristem at different times in a well-ordered sequence. Our model can be readily extended to include the growth of the domain and study precisely this sequential transformation. This extension is currently under investigation and it will be the subject of future publications.
